# Inflammatory Markers in the Blood of Spastic Cerebral Palsy Children: A Case–Control Study

**DOI:** 10.3390/children12030343

**Published:** 2025-03-09

**Authors:** Özlem Tezol, Sıddika Songül Yalçın, Gözde Girgin, Anıl Yirün, Sonia Sanajou, Aylin Balcı Özyurt, Belgin Bayram, Oytun Portakal, Terken Baydar, Çetin Okuyaz, Pınar Erkekoğlu

**Affiliations:** 1 Department of Pediatrics, Faculty of Medicine, Mersin University, 33110 Mersin, Türkiye; 2 Department of Social Pediatrics, Faculty of Medicine, Hacettepe University, 06230 Ankara, Türkiye; siyalcin@hacettepe.edu.tr; 3 Department of Pharmaceutical Toxicology, Faculty of Pharmacy, Hacettepe University, 06230 Ankara, Türkiye; ggirgin@hacettepe.edu.tr (G.G.); soniasanajou@hacettepe.edu.tr (S.S.); tbaydar@hacettepe.edu.tr (T.B.); erkekp@hacettepe.edu.tr (P.E.); 4 Department of Pharmaceutical Toxicology, Faculty of Pharmacy, Çukurova University, 01250 Adana, Türkiye; ayirun@cu.edu.tr; 5 Department of Pharmaceutical Toxicology, Faculty of Pharmacy, Bahçeşehir University, 34353 İstanbul, Türkiye; aylin.balciozyurt@bau.edu.tr; 6 Department of Biochemistry, Faculty of Medicine, Hacettepe University, 06230 Ankara, Türkiye; belginbayram@hacettepe.edu.tr (B.B.); oytun@hacettepe.edu.tr (O.P.); 7 Department of Pediatric Neurology, Faculty of Medicine, Mersin University, 33110 Mersin, Türkiye; okuyazc@mersin.edu.tr

**Keywords:** cerebral palsy, folate, inflammation, kynurenine/tryptophan, neopterin, VitD

## Abstract

Objectives: The aim was to simultaneously investigate inflammatory biomarkers, neopterin, the kynurenine/tryptophan (Kyn/Trp) pathway, vitamin D (VitD), vitamin D binding protein (VDBP), and erythrocyte folate, in cerebral palsy (CP). Methods: A case–control study was conducted at Mersin University Hospital. Three- to ten-year-old patients with spastic CP (n = 50) and age- and gender-matched healthy controls (n = 55) were included. Serum levels of neopterin, Trp, Kyn and 25OHD, plasma VDBP, and erythrocyte folate concentrations were measured. Indoleamine-2,3-dioxygenase 1 (IDO-1) enzyme activity was evaluated according to the Kyn/Trp ratio. Comparison and correlation analyses were performed. Results: The levels of neopterin, Trp, and Kyn were lower in children with CP than in healthy controls (*p* = 0.037, *p* < 0.001, and *p* = 0.003, respectively). IDO1 was not significantly different between the CP and control groups (*p* = 0.214). The levels of VitD and VDBP were higher in children with CP (*p* < 0.001 and *p* = 0.001, respectively). The level of erythrocyte folate was also higher in children with CP (*p* < 0.001). No significant correlation was found between age and inflammatory biomarkers in the CP group. Neopterin was correlated with the level of Gross Motor Function Classification System (GMFCS) level (r = 0.292, *p* = 0.044), while there was no significant correlation between the other biomarkers and the level of GMFCS in the CP group. Conclusions: Inflammatory biomarkers of neopterin and Kyn are lower, inflammatory biomarkers of VDBP and erythrocyte folate are higher, and anti-inflammatory VitD is higher in children with spastic CP compared to healthy children. More knowledge is needed to demonstrate inflammatory and anti-inflammatory status in children with CP.

## 1. Introduction

Cerebral palsy (CP) is defined as “the most common cause of permanent motor disability in children and the result of a non-progressive injury to the immature brain that occurs before birth, during birth, in the neonatal period or by the age of two years” [1]. Cerebral palsy is observed in 2.1/1000 live births in developed countries, with a higher prevalence in low-income countries [1,2,3]. The main symptoms of CP include spasticity (most common), dyskinesia (dystonia, athetosis, chorea), ataxia, and hyperreflexia. Secondary musculoskeletal disorders, epilepsy and sensory, perceptual, cognitive, behavioral, and communication disorders often accompany the motor disorders of CP [4]. The most common type of CP, according to neuro-anatomical classification, is the spastic type, which occurs in more than 80% of cases [5].

Both acute and chronic inflammation have been implicated in fetal or neonatal brain damage contributing to impaired brain development. While inflammation alone is not considered the primary pathogenic factor in CP, growing evidence suggests that a sustained inflammatory response may play a role in its progression [6,7]. According to the “sustained inflammation hypothesis”, neuroinflammatory processes initiated by perinatal insults may persist as a comorbidity in CP, leading to chronic alterations in immune activation and inflammatory biomarker levels. This persistent inflammation is thought to be mediated through various molecular and cellular cascades, contributing to ongoing neuroimmune dysregulation in CP patients [8,9].

Despite theoretical knowledge of chronic inflammation in CP, there is a lack of clinical data on specific inflammatory biomarkers that may characterize this condition. Previous studies have identified elevated levels of neopterin, kynurenine (Kyn), vitamin D binding protein (VDBP), and erythrocyte folate, along with reduced levels of vitamin D (VitD), in various inflammatory and neurological conditions [10,11,12,13,14,15,16,17,18,19,20]. Neopterin is considered as a sensitive biomarker of cell-mediated immune system activation. The concentration of neopterin in biological fluids is increased during inflammation under a wide range of conditions [10,11]. Tryptophan (Trp) is an essential amino acid that effects neuronal function and inflammatory responses. The major Trp metabolism pathway is the Kyn pathway, and this plays a role in inflammation. Indoleamine-2,3-dioxygenase 1 (IDO1) is the rate-limiting enzyme in the Kyn pathway, and inflammatory stimulation leads to increased IDO1 expression. The Kyn/Trp ratio is an indicator of IDO enzyme activity. The level of Kyn and the plasma Kyn/Trp ratio are highly correlated with the clinical features and adverse clinical outcomes of various inflammatory diseases [12,13]. The relationships between B vitamins and inflammatory biomarkers have been reported, suggesting an interaction between one-carbon metabolism and inflammation. However, findings from different studies about the relationship between erythrocyte folate and inflammation are contradictory [14,15]. VitD is traditionally known as a vitamin that regulates bone homeostasis. VitD also has many extraskeletal effects, including the modulation of inflammatory and immune responses [16]. VitD upregulates anti-inflammatory mediators and downregulates inflammatory mediators. Many epidemiologic studies have reported a negative correlation between the blood levels of 25-hydroxy vitamin D (25OHD, the true precursor of the active hormone calcitriol) and inflammatory disease parameters [16,17]. VDBP has potential immunoregulatory functions in addition to VitD binding [18]. VDBP has been found to be positively correlated with inflammatory biomarkers, and a significant association between higher VDBP concentration and exacerbations of inflammatory disease has been reported [19,20].

Recent systematic reviews have identified inflammatory biomarkers in children with CP and emphasized that there is still limited literature on the longitudinal course of inflammatory biomarkers after the acute phase of brain injury in children with CP [8,9]. Although there are a few studies that have examined the use of neopterin and the Kyn/Trp ratio as biomarkers for neuroinflammation, most research has focused on these biomarkers in isolation rather than collectively [21,22]. Yan et al. [21] investigated these biomarkers in cerebrospinal fluid (CSF) within the context of active neuroinflammation, including cases of chronic progressive conditions such as CP, highlighting the diagnostic utility of neopterin, Kyn, quinolinic acid, and the Kyn/Trp ratio. Similarly, Carreras et al. [22] examined the role of CSF neopterin and β2-microglobulin in hypoxic–ischemic encephalopathy (HIE), linking elevated levels of these markers to the severity of brain injury and neurodevelopmental outcomes. These findings underscore the potential of these inflammatory biomarkers not only in diagnosing neuroinflammation but also in monitoring disease progression and evaluating therapeutic interventions in various neurological disorders. For instance, CP is usually associated with epilepsy and neuroinflammation is considered to be a primary factor in epileptogenesis. So, inflammatory biomarkers may play a role in individualizing and monitoring anti-epileptic treatment, based on the CP patients’ biomarker profiles [23].

To the best of our knowledge, neopterin, the Kyn/Trp pathway, VitD, VDBP, and erythrocyte folate are under-researched biomarkers in CP with regard to inflammation. We hypothesize that the combined analysis of these biomarkers will provide a more comprehensive understanding of inflammation in children with CP. Given the potential relevance of inflammation in CP, this study aims to comprehensively evaluate a panel of inflammatory biomarkers (neopterin, Kyn/Trp ratio, VitD, VDBP, and erythrocyte folate) in children with spastic CP compared to healthy controls. Additionally, as epilepsy is a common comorbidity in CP and may be linked to neuroinflammation, we also investigated potential differences in these biomarkers based on the presence of epilepsy in children with CP. Our findings may contribute to a better understanding of the inflammatory mechanisms in CP, potentially aiding in the identification of novel biomarkers for disease monitoring and therapeutic targets.

## 2. Materials and Methods

### 2.1. Study Design and Participants

This case–control study was conducted at Mersin University Hospital. Patients with spastic CP and age- and gender-matched healthy controls formed the experimental group of this study. Three- to ten-year-old patients followed up in the Pediatric Neurology clinic and healthy children admitted to the well child outpatient clinic were included in this study. Patients with dyskinetic, ataxic, or mixed-type CP, neuromuscular disorders other than CP, a feeding tube and/or tracheostomy, congenital malformations, multiple organ dysfunction, or infectious diseases were excluded. Exclusion criteria also included the use of botulinum neurotoxin A injections and/or vitamin supplements within six months prior to blood sampling. The Mersin University Local Ethics Committee approved the study protocol (2020/312).

### 2.2. Sample Collection

A study file was developed and administered to parents to collect data about sociodemographic characteristics. The income level of the family and type of residence were categorized according to the Turkish Statistical Institute classifications [24]. Anthropometric measurements and general physical examinations were performed in all subjects. Weight-for-age (WFA), height-for-age (HFA), and body mass index (BMI)-for-age z-scores were calculated using the child growth standards of the World Health Organization [25,26]. Clinical characteristics (type of spastic CP, level of Gross Motor Function Classification System (GMFCS) [27]) and comorbidities were recorded.

Blood samples were collected from the antecubital vein of CP patients and healthy children in the early morning. Plasma and serum samples were separated and stored at −80 °C until analysis.

### 2.3. Measurement of Biomarkers

Serum level of neopterin was determined by an enzyme immunoassay using a commercially available neopterin assay (Human Serum Neopterin Assay, EIA-1476, DRG Instruments GmbH, Marburg, Germany) according to the manufacturer’s instructions. Absorbance was read at 450 nm using a microplate reader. Serum level of neopterin was represented in nmol/L.

Serum levels of Trp and Kyn were measured by the HP Agilent 1100 HPLC system (Agilent, Vienna, Austria) using a reversed-phase Hichrom C18 column (5 µm, 25 cm × 4.6 mm i.d., Hichrom Ltd., Berkshire, UK). The proteins were precipitated with perchloric acid. The level of Trp was determined through natural fluorescence detection (G1312A; Agilent) at λ_excitation_ = 285 nm and λ_emission_ = 365 nm. On the other hand, the level of Kyn was determined through variable wavelength detection (G1314A; Agilent) using 360 nm wavelength ultraviolet (UV) absorption [28,29]. The levels of both Trp and Kyn were expressed in µmol/L. IDO-1 enzyme activity was evaluated according to the serum levels of Trp and Kyn and the Kyn/Trp ratio. IDO-1 activity was given in µmol/mmol.

Serum total 25OHD concentrations were measured by a direct competitive enzyme-linked immunosorbent assay (ELISA) using 25-HVD3 (25-Hydroxy vitamin D3) ELISA Kit (ElabScience, Houston, TX, USA) and expressed in ng/mL. The detection range of the ELISA kit was 3.13–200 ng/mL, and the sensitivity was 1.88 ng/mL. The vitamin D status of the participants was classified as follows: sufficient > 20 ng/mL, insufficient 12–20 ng/mL, and deficient < 12 ng/mL [30].

Measurement of circulating plasma VDBP concentration was carried out with sandwich ELISA using a human DBP (vitamin D binding protein) ELISA Kit (ElabScience, Houston, TX, USA) according to the manufacturer’s instructions and expressed in mg/mL. The detection range of the kit was 3.91–250 ng/mL, and the sensitivity was 2.35 ng/mL.

Erythrocyte folate concentrations were determined by a chemiluminescence immunoassay with paramagnetic particles (Beckman Coulter, Inc., Brea, CA, USA) and expressed in ng/mL. Folate deficiency was defined as an erythrocyte folate < 140 ng/mL. Marginal folate deficiency was defined as an erythrocyte folate between 140 and 200 ng/mL. Folate insufficiency was defined as an erythrocyte folate ≤ 200 ng/mL [31].

### 2.4. Sample Size

All patients with spastic CP who were admitted to the Child Neurology outpatient clinic between 1 October and 30 December 2020, fulfilled the appropriate criteria, and agreed to participate in this study constituted the study sample. For each CP patient, an age- and gender-matched healthy child with normal growth and development was also included in this study. In total, data were collected from 68 children with spastic CP and 68 healthy children. In addition, 8 CP patients with active epilepsy, 10 CP patients with inadequate or inappropriate blood sample volume, and 13 healthy children with inadequate or inappropriate blood sample volume were excluded from the analysis.

### 2.5. Statistical Analysis

SPSS version 26.0 software (IBM Corp., Armonk, NY, USA) was used to determine significant differences between the CP and healthy control groups. The Shapiro–Wilk test and histograms were used to test the normality of the data. The results are expressed as mean ± standard deviation (SD), median (IQR, 25th–75th percentile), or number (percentage, %). The chi-square test was used for categorical variables. Student’s *t*-test, the Mann–Whitney U test, and the Kruskal–Wallis test were used to compare quantitative variables between independent groups. Spearman’s rank correlation was used to measure the relationship between the biomarkers. Generalized linear models were used to compare differences in each biomarker after adjusting for child age, gender, prematurity, childcare provider, family income level, z-score for HFA, and z-score for BMI and means with 95% confidence intervals were calculated. Skewed data were analyzed with a gamma-log link model. The statistical significance level was taken as *p* < 0.05.

## 3. Results

The etiology of CP was preterm birth in 30 patients (60%) and HIE in 20 patients (40%). Spastic quadriplegia, spastic diplegia, and spastic hemiplegia were found in 26 (52%), 14 (28%), and 10 (20%) patients, respectively. Thirteen (26%), six (12%), ten (20%), and twenty-one (42%) patients were diagnosed with GMFCS level II, GMFCS level III, GMFCS level IV, and GMFCS level V, respectively. Nineteen patients (38%) were ambulant and thirty-one patients (62%) were non-ambulant. Twenty patients (44%) had stable epilepsy and antiepileptic use. All patients consumed calorically dense enteral nutrition solution daily.

Sociodemographic and anthropometric characteristics are shown in Table 1. Preterm birth, maternal care, and low family income were significantly more common in children with CP (*p* < 0.05). Mean WFA, HFA, and BMI z-scores were significantly lower in the CP group (*p* < 0.05).

Table 2 shows the comparison of the concentration of biomarkers between the CP and healthy control groups. The levels of neopterin, Trp, and Kyn were lower in children with CP than in healthy controls (*p* = 0.037, *p* < 0.001, and *p* = 0.003, respectively). IDO1 was not significantly different between the CP and control groups (*p* = 0.214). The levels of VitD and VDBP were higher in children with CP (*p* < 0.001 and *p* = 0.001, respectively). The level of erythrocyte folate was also higher in children with CP (*p* < 0.001).

Table 3 presents the comparison of inflammatory biomarkers between the CP and healthy control groups after adjustment for confounding factors. The levels of neopterin, Trp, and IDO1 were not different between the CP and control groups (*p* > 0.05), whereas the levels of Kyn, VDBP, VitD, and erythrocyte folate were significantly higher in the CP group after adjusting for confounding factors (*p* < 0.05).

Correlations between biomarkers and age are presented in Table 4. A weak correlation was found between age and the level of VDBP in healthy controls (*p* = 0.020). No significant correlation was found between age and biomarkers in the CP group. In the CP group, neopterin was weakly negatively correlated with Trp and VDBP (*p* = 0.019 and *p* = 0.007, respectively); Trp was moderately correlated with Kyn (*p* < 0.001); Kyn was strongly correlated with IDO1 (*p* < 0.001). In the control group, neopterin was moderately correlated with Kyn and IDO1 (*p* = 0.001 and *p* < 0.001, respectively); neopterin was weakly negatively correlated with VDBP (*p* = 0.041); Trp was weakly correlated with Kyn and VDBP (*p* = 0.007 and *p* = 0.032); Kyn was strongly correlated with IDO1 (*p* < 0.001); IDO1 was weakly negatively correlated with VDBP (*p* = 0.008).

Anthropometric z-scores were not significantly correlated with inflammatory biomarkers (Table 4). Neopterin was correlated with the level of GMFCS (r = 0.292, *p* = 0.044), while there was no significant correlation between the other biomarkers and the level of GMFCS in the CP group (Table 4).

Table 5 and Supplementary Figure S1 show the comparison of inflammatory biomarkers between the CP with epilepsy, CP without epilepsy, and healthy control groups. The level of neopterin was significantly lower in the CP children without epilepsy than in the CP children with epilepsy and healthy controls (*p* = 0.024). The level of Trp was significantly higher in the healthy controls than in the CP children with and without epilepsy (*p* = 0.001). The level of Kyn was not different between the healthy controls and CP children without epilepsy, while it was significantly higher in healthy controls than in the CP children with epilepsy (*p* = 0.003). The levels of VitD, VDBP, and erythrocyte folate were significantly lower in the healthy controls than in the CP children with and without epilepsy (*p* = 0.003, *p* < 0.001, and *p* = 0.001, respectively). IDO1 was not significantly different between the CP with epilepsy, CP without epilepsy, and healthy control groups (*p* = 0.110). Frequencies of VitD insufficiency/deficiency and erythrocyte folate insufficiency were not different between the CP with epilepsy, CP without epilepsy, and healthy control groups (*p* = 0.418 and *p* = 0.241, respectively).

## 4. Discussion

In this study, peripheral blood samples were measured, and a total of six biomarkers of inflammation were examined together for the first time. It has been concluded that cytokine responses, distribution, and frequency of adaptive and innate immune cells, and subsequent systemic inflammatory status are altered in children with CP [9]. Plasma or serum TNFα, IL-6, and IL-10 were the most commonly investigated inflammatory biomarkers, and conflicting findings have been reported [9]. Although different levels of inflammatory biomarkers have been documented in CP compared to controls, the orientation appears to be mixed [9]. Our findings suggest that inflammatory biomarkers of neopterin and Kyn are lower, inflammatory biomarkers of VDBP and erythrocyte folate are higher, and anti-inflammatory VitD is higher in children with CP compared to healthy children.

Pingel et al. [32] investigated the blood level of systemic inflammatory markers in children and adults with CP compared to healthy adults. The authors showed that systemic transforming growth factor beta 1 (TGFβ1) and C-reactive protein (CRP) levels were significantly higher in school-age children with CP compared to both adults with CP and healthy adults and suggested an increased level of systemic inflammation in 10.4 ± 1.1-year-old children with CP [32]. The present study revealed a decreased level of systemic inflammation in children with CP aged between 3 and 10 years compared to their healthy peers. Pingel et al. [32] included pediatric CP patients, 93% of whom were ambulant and 93% were hemiplegic or diplegic [32]. However, the majority of our subjects were tetraplegic and non-ambulant CP patients. These differences about CP may have led to differences in individual and environmental characteristics, and different physiological, social, environmental, and lifestyle characteristics may have influenced the inflammatory status [33].

Neopterin is a peripheral early biomarker of the cellular immune response. An elevated level of neopterin in CSF is considered as a useful marker of an acute or active CNS injury [34]. Previously, the measurement of neopterin has been used in the clinical laboratory diagnosis of cerebral infections and inflammatory and auto-immune CNS disorders in children [29,35]. Dale et al. [35] reported that CSF neopterin is a marker of an active or degenerative process, not a static process, because they found that the level of neopterin was predominantly low in children with chronic static CNS disorders, including CP. In our study, the level of neopterin was not different between the CP and control groups after adjusting for confounding factors. As highlighted by Dale et al. [35], the lack of elevated level of neopterin may suggest that inflammation is rare in patients with CP. The effect of environment, nutrition, and lifestyle can be investigated in further studies because the microbiome, food and beverages consumed, or physical exercise may affect the formation of neopterin [36]. In our study, neopterin showed a weak correlation with the level of GMFCS. Previously, plasma levels of TNF were reported to be correlated with a higher level of GMFCS only in CP patients with spastic diplegia, whereas no correlation was observed when looking at the whole CP group [37]. Therefore, although findings are variable, CP severity may be associated with certain increased inflammatory biomarkers.

Tryptophan metabolism plays an important role in the control of inflammation. The IDO enzyme controls inflammation in response to unexpected stimuli, and Trp metabolites are involved in the control of neuronal inflammation [38]. Kynurenine plays a role in the pathogenesis of brain disorders, and the Kyn pathway has become a subject of research in neuropsychiatric and neurodegenerative disorders (e.g., schizophrenia, Huntington’s disease, Alzheimer’s disease, Parkinson’s disease) [39,40]. Since the expression of IDO1 is induced by pro-inflammatory cytokines, Kyn and the Kyn/Trp ratio have been reported to be useful biomarkers of both systemic inflammatory and neuroinflammatory conditions in children [39,40,41,42,43,44,45,46]. In this study, the levels of Trp and Kyn were lower in the CP group. Since Trp is an essential amino acid derived primarily from the diet, Trp availability may be reduced in the diets of children with CP, who have significantly lower anthropometric z-scores. Trp degradation at the Kyn axis is susceptible to modulation by nutrition, exercise, microbiome, and stress [36]. Differences in these personal and environmental factors may be the reason for lower Kyn in children with CP than in healthy children. Furthermore, IDO1 has been suggested to play a role in the pathogenesis of epilepsy [47]. Epilepsy and antiepileptic therapeutics have been shown to affect the Trp/Kyn axis [48,49]. Therefore, the presence or absence of epilepsy and antiepileptic therapies in children with CP may affect inflammatory marker levels. Our study also revealed that Trp and Kyn levels significantly differed between CP patients with stable epilepsy and their healthy peers.

In a recent study, compared to control groups, statistically significant increases in CSF neopterin, Kyn, and the Kyn/Trp ratio were found in pediatric patients with primary neuroinflammatory disorders, and they have been reported to be useful diagnostic and monitoring biomarkers of neuroinflammation [21]. We found statistically significant decreases in serum neopterin and Kyn in pediatric patients with spastic CP, which is generally considered a non-progressive (“static”) disorder.

Folate is a B vitamin derived from natural sources and acts as a cofactor for one-carbon transfer reactions involved in the development and maintenance of inflammatory responses. There is an association between folate and inflammation, as the optimal level of folate can prevent endothelial dysfunction and DNA instability. Low levels of folate are commonly observed in chronic inflammatory diseases, indicating that insufficient folate may play a role in the pathogenesis of inflammatory conditions or that chronic inflammation increases folate requirements [50]. Erythrocyte folate is a good indicator of folate status, and biomarkers of inflammation in women have been associated with erythrocyte folate [14]. In contrast, no significant correlation between inflammation and erythrocyte folate has been reported in children. In a study conducted among preschool children, a weak and significant negative correlation (−0.07) was found between erythrocyte folate and the inflammation biomarker CRP, while in another study, no significant correlation was found between these two parameters in children [15,51]. In this study, we did not find a correlation between the levels of erythrocyte folate and other inflammatory biomarkers, and we observed a similar erythrocyte folate status in children with spastic CP and their healthy peers. Both previous studies [15,51] and our study suggest that the relation between folate status and inflammation may vary based on the severity of folate deficiency and the underlying prevalence of inflammation. Varying laboratory methods may also impair building a consensus on which associations exist between folate and inflammation indicators [15].

Since VitD reduces chronic inflammation, an inverse relationship between 25OHD and clinical manifestations of inflammatory diseases has been shown [16]. VitD status is also associated with inflammation in childhood and adolescence. Previously, 25(OH)D concentrations have been reported to be inversely associated with the level of inflammatory biomarkers such as IL-6, cathepsin S, and vascular cell adhesion molecule-1, while some studies have not found a relationship between the concentrations of 25(OH)D and CRP in children and adolescents [17]. We found no correlations between serum 25(OH)D levels and levels of inflammatory biomarkers. Some authorities believe that low 25(OH)D is a consequence of chronic inflammation rather than the cause [52], and it is known that children with CP are a higher risk group for low VitD. Thus, certain low VitD levels in CP children may be further discussed in detail in terms of chronic inflammation beyond nutrition. In a recent study conducted in Turkey, adequate VitD levels were reported in 57% of children with CP [53]. In our study, this rate was 65%, and we observed higher VitD levels in children with spastic CP compared to the healthy controls. Daily enteral nutrition solution consumption may have provided a better VitD status in our patients with CP.

The role of VDBP in modulating the immune and inflammatory response is less known than its functions such as the transport of VitD metabolites, binding and transport of fatty acids, and the scavenging of actin. VDBP plays an important role in maintaining microcirculation, inflammation, and infection [54]. Serum VDBP levels have been correlated with inflammatory biomarkers such as erythrocyte sedimentation rate, transferrin, and high-sensitivity CRP [20]. Also, a significant correlation between the serum level of VDBP and anti-inflammatory cytokines has been detected [55]. We found an inverse correlation between VDBP and neopterin in both of the CP and control groups. Furthermore, VDBP was negatively correlated with IDO1 activity in the healthy controls. Although our findings support that higher levels of inflammation may be associated with lower serum VDBP in children including those with CP, there is a need for an international standard for (gene-specific) VDBP and a reference method for the measurement of VDBP in systemic and local inflammatory conditions [56]. Furthermore, considering that VDBP is the most polymorphic protein known [54], whether and how inflammation affects the regulation of VDBP in CP individuals should be examined in further studies.

Previously, an age-related difference in inflammatory status in children with CP has been reported. Younger CP children (1–3 years) have been reported to have more systemic inflammation [significantly increased plasma TNFα] compared to older CP children (4–12 years) [35]. In addition, no significant inflammatory difference was found in plasma CRP between adults with CP and healthy adults [32]. These findings may indicate that inflammatory status changes over time and becomes less pronounced with increasing age [9]. However, this study did not show any correlation between age and the level of inflammatory biomarkers in 3- to 10-year-old children with CP. More research is needed to demonstrate longitudinal inflammatory and anti-inflammatory status in CP individuals. As a predictor of increased IDO activity, the Kyn/Trp ratio was found to be negatively associated with linear growth in children from Bangladesh [57]. However, this study found no correlation between anthropometric z-scores and Trp, Kyn, or IDO1. The variation in Trp and Kyn or Kyn/Trp ratio according to nutritional status and how it changes could be further investigated in prospective CP studies.

The severity, topography, and etiology of CP, its comorbidities and medications, genetic polymorphisms, and epigenetic changes may affect inflammatory status [9]. The heterogeneity of our patients in these aspects is a limitation of the present study. The stable epilepsy of the CP participants indicating no uncontrolled seizures and not including children consuming dietary supplements may alleviate this limitation of this study. Other limitations of this study are the fact that it is a hospital-based single-center study and dietary characteristics and environmental exposures of children were not addressed in detail. Nevertheless, in terms of the role of VitD, we can say that our previous study [58] revealed that daily unstructured, free time outside did not differ between our CP sample and healthy control group. Future studies with multicenter design and larger sample sizes may be conducted to investigate the alterations in inflammatory biomarkers. Acute and long-term changes in various inflammatory biomarkers in response to dynamic standing exercise have been reported in immobilized children with CP [59]. However, we did not consider the regular exercise and physical therapy status of patients with CP. Further research analyzing inflammatory biomarker levels is needed to understand clinical–biological correlations in CP. Although our study does not directly contribute the detection, monitoring, or treatment of CP, our findings may provide insight into future research into novel diagnostic and therapeutic options.

## 5. Conclusions

The present findings indicate that serum levels of neopterin, Trp, and Kyn were lower, whereas the levels of VitD, VDBP, and erythrocyte folate were higher in children with spastic CP. The findings on Kyn, VitD, VDBP, and erythrocyte folate remained significant after adjusting for potential confounders. We demonstrate that these biomarkers of inflammation, which are understudied in CP, were altered in spastic CP children aged 3–10 years. Still, more knowledge is needed to determine inflammatory and anti-inflammatory status in children with CP.

## Figures and Tables

**Table 1:** Sociodemographic and anthropometric characteristics by spastic CP and healthy control groups.

	Cerebral Palsy (n = 50)	Healthy Controls (n = 55)	*p*
Age	6.8 (5.0–8.3)	7.3 (5.3–8.9)	0.552 ^b^
Gender, male	27 (54.0)	27 (49.1)	0.615 ^c^
Type of delivery, cesarean	38 (76.0)	34 (61.8)	0.118 ^c^
Gestational age, preterm birth	35 (70.0)	0 (0)	<0.001 ^c^
Appropriate for gestational age	47 (94.0)	51 (92.7)	1.00 ^c^
Birth order			0.564 ^c^
1st	18 (36.0)	24 (43.6)	
2nd	20 (40.0)	22 (40.0)	
≥3rd	12 (24.0)	9 (16.4)	
Childcare provider			
Mother	48 (96.0)	46 (83.6)	0.039 ^c^
Grandmother, caretaker, or kindergarten	2 (4.0)	9 (16.4)	
Maternal age, years	35.1 ± 5.4	33.4 ± 5.5	0.107 ^d^
Paternal age, years	39.6 ± 6.4	38.4 ± 5.9	0.305 ^d^
Maternal educational level of ≥8 years ^a^	17 (34.0)	24 (43.6)	0.312 ^c^
Paternal educational level of ≥8 years ^a^	20 (40.0)	31 (56.4)	0.094 ^c^
Maternal smoking	8 (16.0)	9 (16.4)	0.960 ^c^
Paternal smoking	25 (50.0)	25 (45.5)	0.641 ^c^
Tobacco smoke exposure	11 (22.0)	18 (32.7)	0.220 ^c^
Number of children in the family	2 (2–3)	2 (2–3)	0.142 ^b^
Family structure, nuclear	41 (82.0)	46 (83.6)	0.824 ^c^
Family income level			0.048 ^c^
High	5 (10.0)	16 (29.1)	
Middle	18 (36.0)	17 (30.9)	
Low	27 (54.0)	22 (40.0)	
Settlement, urban/semi-urban	46 (92.0)	51 (92.7)	1.00 ^c^
Z-score			
Weight for age	−1.07 ± 1.95	0.12 ± 1.21	<0.001 ^d^
Height for age	−1.13 ± 1.75	−0.10 ± 1.05	0.001 ^d^
Body mass index for age	−0.60 ± 2.08	0.23 ± 1.21	0.016 ^d^

^a^ More than or equal to elementary level of education. ^b^ Mann–Whitney U test; data are presented in median (IQR). ^c^ Chi-square test; data are presented in numbers (percentages). ^d^ Student’s *t*-test; data are presented in mean ± SD.

**Table 2:** Comparison of inflammatory biomarkers between spastic CP and healthy control groups.

	Cerebral Palsy (n = 50)	Healthy Controls (n = 55)	*p*
Neopterin, nmol/L	8.10 ± 2.17	9.09 ± 2.43	0.037 ^a^
	[7.86 (7.10–9.23)]	[8.85 (7.37–11.47)]	
Tryptophan, µmol/L	47.29 ± 8.25	53.33 ± 7.95	<0.001 ^a^
	[47.37 (43.22–51.60)]	[53.01 (48.52–57.84)]	
Kynurenine, µmol/L	2.22 (1.81–2.48)	2.60 (2.21–2.93)	0.003 ^b^
	(2.29 ± 0.75)	(2.58 ± 0.63)	
IDO1, µmol/mmol	44.94 (40.14–50.12)	47.34 (43.42–52.31)	0.214 ^b^
	(49.66 ± 24.24)	(48.88 ± 13.09)	
VDBP, mg/mL	0.38 (0.35–0.42)	0.31 (0.28–0.36)	<0.001 ^b^
	(0.43 ± 0.14)	(0.35 ± 0.13)	
Vitamin D, ng/mL	23.6 (18.3–33.4) ^&^	18.8 (16.8–22.6)	0.001 ^b^
	(28.3 ± 14.1)	(20.2 ± 6.0)	
Sufficient	32 (65.3)	24 (43.6)	0.038 ^c^
Insufficient	17 (34.7)	29 (52.7)	
Deficient	0 (0)	2 (3.6)	
Erythrocyte folate, ng/mL	409 (352–451) ^#^	350 (307–383) ^&^	<0.001 ^b^
	(415 ± 95)	(346 ± 73)	
Sufficient	45 (97.8)	44 (89.8)	0.205 ^c^
Insufficient	1 (2.2)	5 (10.2)	
Deficient	0 (0)	0 (0)	

^a^ Student’s *t*-test; data are presented in mean ± SD. ^b^ Mann–Whitney U test; data are presented in median (IQR). ^c^ Chi-square test; data are presented in numbers (percentages). IDO1: indoleamine 2,3-dioxygenase 1; VDBP: vitamin D binding protein. ^&^ n = 49; ^#^ n = 46.

**Table 3:** Comparison of inflammatory biomarkers between spastic CP and healthy control groups after adjusting confounding factors ^a^.

	Cerebral Palsy (n = 50)	Healthy Controls (n = 55)	*p*
Neopterin (nmol/L)	7.88 [6.85–8.93]	8.88 [7.88–9.89]	0.148
Tryptophan (µmol/L)	48.4 [44.9–51.8]	51.3 [47.9–54.6]	0.210
Kynurenine (µmol/L)	2.19 [1.88–2.51]	2.66 [2.36–2.96]	0.026
IDO1 (µmol/mmol)	45.6 [40.0–51.2]	52.3 [46.9–57.7]	0.075
VDBP (mg/mL)	0.44 [0.38–0.51]	0.35 [0.31–0.40]	0.007
Vitamin D (ng/mL)	27.4 [23.2–32.3] ^&^	18.2 [15.6–21.4]	<0.001
Erythrocyte folate (ng/mL)	416 [376–461] ^#^	358 [324–395] ^&^	0.031

^a^ Mean [95% Wald CI]; adjusted for age, gender, prematurity, childcare provider, family income level, z-score for height for age, and z-score for body mass index for age. IDO1: indoleamine 2,3-dioxygenase 1; VDBP: vitamin D binding protein. ^&^ n = 49; ^#^ n = 46.

**Table 4:** Correlations between inflammatory biomarkers.

		Neopterin	Tryptophan	Kynurenine	IDO1	Erythrocyte Folate	VDBP	VitD
Age	CP	−0.218	−0.167	0.045	0.179	0.105	−0.008	−0.135
	Control	−0.110	0.065	−0.038	−0.072	−0.097	0.319 *	0.032
Neopterin	CP		−0.338 *	−0.230	−0.053	0.055	−0.383 **	0.036
	Control		0.014	0.471 **	0.512 **	0.217	−0.296 *	0.138
Tryptophan	CP			0.502 **	−0.075	−0.220	0.020	0.070
	Control			0.359 **	−0.209	−0.150	0.295 *	−0.096
Kynurenine	CP				0.736 **	−0.018	−0.062	−0.062
	Control				0.748 **	−0.181	−0.263	0.050
IDO1	CP					0.129	−0.114	−0.076
	Control					0.049	−0.360 **	0.149
Erythrocyte	CP						−0.101	−0.071
folate	Control						0.085	0.066
VDBP	CP							0.210
	Control							−0.114
Weight-for-age z-score	CP	−0.114	−0.104	0.026	0.129	−0.103	−0.082	−0.114
	Control	0.199	0.210	0.157	0.022	−0.123	−0.041	0.199
Height-for-age z-score	CP	0.078	−0.240	0.013	0.126	−0.185	−0.101	0.078
	Control	0.299	0.110	0.170	0.087	−0.106	−0.143	0.299
BMI-for-age z-score	CP	−0.123	0.050	0.029	0.074	−0.023	−0.029	−0.123
	Control	0.095	0.212	0.117	−0.031	−0.081	0.024	0.095
GMFCS level	CP	0.292 *	−0.273	−0.186	−0.013	0.240	−0.055	0.088

* *p* < 0.05, ** *p* < 0.01 (2-tailed); Spearman’s rho. IDO1: indoleamine 2,3-dioxygenase 1; VDBP: vitamin D binding protein; BMI: body mass index; GMFCS: Gross Motor Function Classification System.

**Table 5:** A comparison of inflammatory biomarkers between the CP with epilepsy, CP without epilepsy, and healthy control groups.

	CP with Epilepsy (n = 20)	CP without Epilepsy (n = 30)	Healthy Controls (n = 55)	*p*
Neopterin (nmol/L)	8.85 (7.31–9.64) ^a^	7.68 (6.72–8.44) ^b^	8.85 (7.37–11.47) ^a^	0.024
Tryptophan (µmol/L)	45.77 (41.52–49.05) ^b^	47.82 (43.23–55.59) ^b^	53.01 (48.52–57.84) ^a^	0.001
Kynurenine (µmol/L)	1.99 (1.60–2.33) ^b^	2.30 (2.05–2.53) ^ab^	2.60 (2.21–2.93) ^a^	0.003
VDBP (mg/mL)	0.38 (0.34–0.43) ^b^	0.38 (0.35–0.41) ^b^	0.31 (0.28–0.36) ^a^	<0.001
Vitamin D (ng/mL)	22.9 (18.3–33.4) ^b^	24.1 (18.3–33.1) ^b,¶^	18.8 (16.8–22.6) ^a^	0.003
Erythrocyte folate (ng/mL)	412 (375–470) ^b^	403 (348–436) ^b,#^	350 (307–383) ^a,&^	0.001

*p*-values are from the Kruskal–Wallis test; data are presented in median (IQR). Values having different letters were found to be statistically different. ^¶^ n = 29, ^#^ n = 26, ^&^ n = 49.

## Data Availability

The data presented in this study are available on request from the corresponding author due to ethical reasons.

## Supplementary Materials

The following supporting information can be downloaded at https://www.mdpi.com/article/10.3390/children12030343/s1, Figure S1: A comparison of inflammatory biomarkers between the CP with epilepsy, CP without epilepsy, and healthy control groups; A: neopterin, B: tryptophan, C: kynurenine, D: vitamin D binding protein (VDBP), E: vitamin D, and F: erythrocyte folate.
