# Supplementary material for: Inflammatory Markers in the Blood of Spastic Cerebral Palsy Children: A Case–Control Study
Source: Children (Basel). 2025 Mar 9;12(3):343. doi: 10.3390/children12030343 (PMC11940874; doi:10.3390/children12030343)
Supplement: Supplementary file 1 [file children-12-00343-s001.zip › children-3468011-supplementary.pdf]

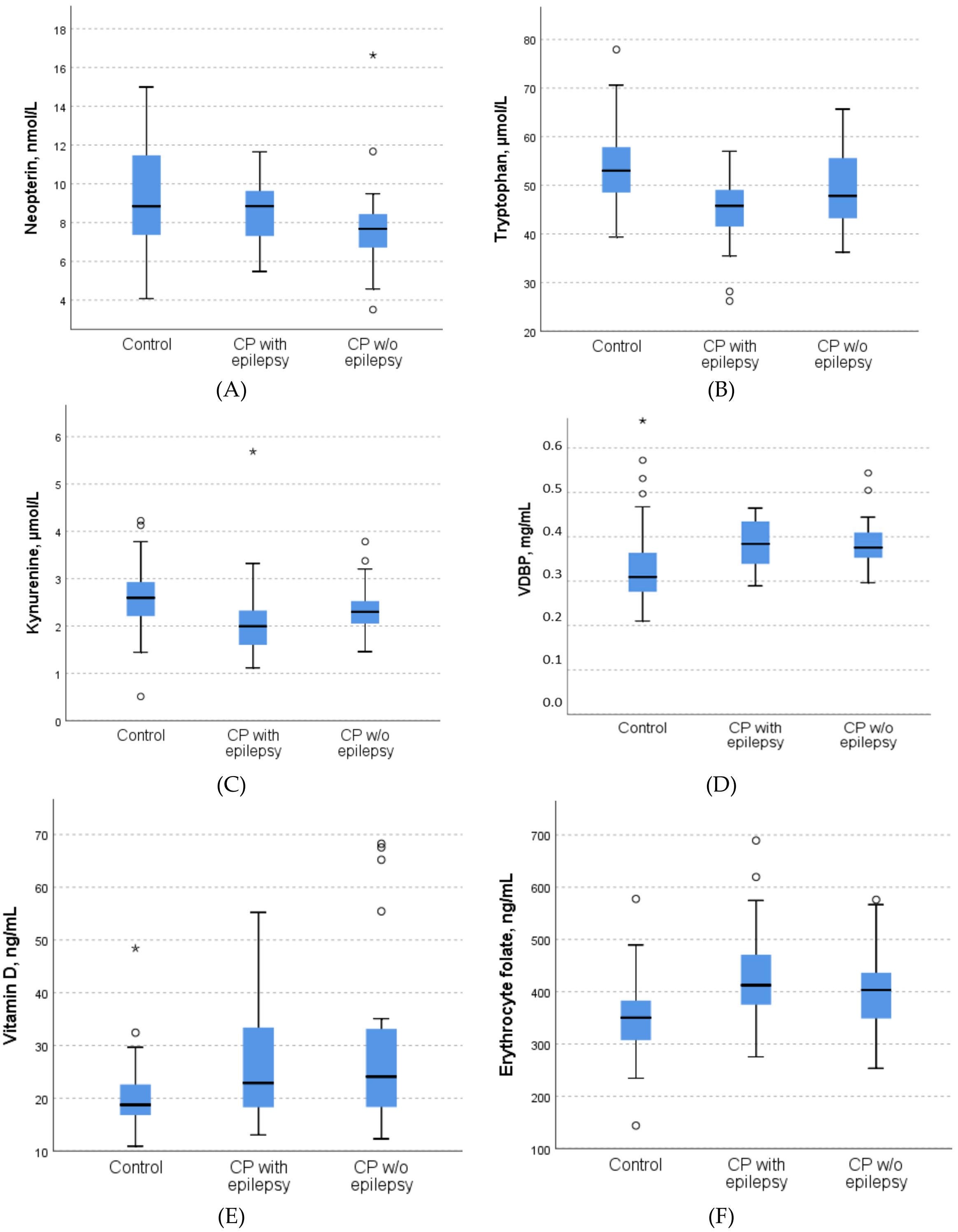

Supplementary Figure S1. The comparison of inflammatory biomarkers between the CP with epilepsy, CP without epilepsy and healthy control groups; A: neopterin, B: tryptophan, C: kynurenine, D: vitamin D binding protein (VDBP), E: Vitamin D, and F: erythrocyte folate.

 https://doi.org/10.3390/children12030343
